# Routine long head of the biceps release improves pain and functional outcomes after arthroscopic rotator cuff repair of degenerative tears: A retrospective comparative study

**DOI:** 10.1051/sicotj/2026018

**Published:** 2026-05-06

**Authors:** Dimitrios V Papadopoulos, Athanasios Kontogiannis, James R. Mullen, Ioannis Papakammenos, Nikolaos Stavropoulos, Konstantinos Tsikopoulos, Ioannis Gkiatas, Vasileios S Nikolaou, George Babis

**Affiliations:** 1 2nd Department of Orthopaedic Surgery, “Konstantopouleio” General Hospital, National and Kapodistrian University of Athens 14233 Athens Greece; 2 St. Clair Health Pittsburgh PA USA; 3 Department of Orthopaedics, School of Medicine, University of Ioannina Ioannina Greece

**Keywords:** Long head of the biceps tendon, Rotator cuff repair, Arthroscopy, Functional outcomes, Pain reduction, Level of evidence: III

## Abstract

*Introduction*: The optimal management of the long head of the biceps tendon (LHBT) during rotator cuff repair remains controversial, particularly when the tendon appears normal. This study aims to compare the clinical outcomes of arthroscopic rotator cuff repair with and without routine LHBT release. *Methods:* A retrospective study including patients aged >50 years with a repairable rotator cuff tear and documented normal LHBT who underwent arthroscopic surgery was conducted. Patients were divided into two groups: LHBT preservation group (*n* = 113) and LHBT release group (*n* = 110). Postoperative evaluation included the visual analog scale (VAS) for pain, while functional outcomes were assessed by the Constant-Murley score and the American Shoulder and Elbow Surgeons (ASES) scores. Postoperative pain and functional outcomes were compared between the two study groups at 12 and 24 months. *Results*: Groups were comparable in terms of age (*p* = 0.16), sex (*p* = 0.30), rotator cuff tear size (*p* = 0.51), and number of anchors used for the repair (*p* = 0.44). At 24 months, the LHBT release group demonstrated lower VAS score (*p* < 0.001), higher Constant-Murley score (medians: 86 vs 81, *p* < 0.001), and higher ASES score (medians: 90 vs 83, *p* < 0.001). Regression analysis confirmed that LHBT release is independently associated with improved functional outcomes (coefficient = 4.85, *p* < 0.001 for Constant-Murley score; coefficient = 6.66, *p* < 0.001 for ASES score). *Discussion*: The findings of this study indicate that routine LHBT release during rotator cuff repair, even in the absence of macroscopic pathology, is associated with less postoperative pain and superior functional scores.

## Introduction

Although its role in shoulder function is poorly understood, the long head of the biceps tendon (LHBT) is considered a significant stabilizer of the glenohumeral joint, especially in pathological conditions, such as rotator cuff tears (RCTs) [1, 2]. Moreover, LHBT has long been recognized as an important contributor to shoulder pain. Lesions of the LHBT are often associated with rotator cuff pathology, such as impingement or RCT, while isolated lesions rarely occur [3]. Involvement of LHBT pathology in supraspinatus tears ranges from 22% to 78.5% of the cases [4]. LHBT pathology varies from SLAP lesions to tendinitis, subluxation, entrapment, delamination tears, and dislocation from the bicipital groove. All these lesions can cause significant forward shoulder dysfunction and anterior shoulder pain [3, 5]. Due to the high incidence of RCTs and the high frequency of LHBT involvement, it is well established that the LHBT is a considerable source of shoulder pain and disability. Therefore, optimal management of LHBT lesions is imperative.

The most common surgical techniques in LHBT management include biceps tenotomy and tenodesis. These procedures provide similar postoperative functional outcomes, pain alleviation, and adverse events [2, 6]. Furthermore, the analgesic role of biceps tenotomy/tenodesis has long been described as beneficial in patients with irreparable RCTs [5]. However, there is no consensus regarding LHBT management in patients with RCTs when the LHBT appears macroscopically intact. Some authors recommend routine tenotomy or tenodesis with rotator cuff repair, while others support tenotomy/tenodesis only when subscapularis tears are evident, or in the presence of significant pathology of the biceps [5, 7]. Relevant studies provide contradictory results regarding postoperative functional scores (i.e., Constant-Murley and American Shoulder and Elbow Surgeons [ASES] scores) and pain alleviation (i.e., visual analog scale [VAS] score) of RCT repair with routine LHBT release compared to RCT repair without LHBT management [5, 7–9].

The purpose of this retrospective study was to compare the functional outcomes of patients who underwent arthroscopic rotator cuff surgery with retention of normal LHBT, versus those who underwent surgery with additional release of an otherwise normal LHBT. We hypothesize that there would be a significant difference between the two study groups regarding functional outcomes and pain improvement.

## Material and methods

A retrospective comparative observational study was conducted between 09/2020 and 09/2023. Inclusion criteria were patients aged >50 years with a repairable degenerative posterosuperior RCT and documented normal LHBT who underwent arthroscopic surgery. Normal LHBT should have been documented on preoperative imaging and additionally confirmed intraoperatively. Moreover, a minimum postoperative follow-up of 2 years was required. Patients who underwent revision rotator cuff repair or patients with additional pathology such as acromioclavicular joint arthritis, glenohumeral cartilage lesions, and subscapularis tear were excluded. Moreover, patients with incomplete clinical records were excluded. Patients were identified through a review of our clinic’s electronic records and operating room notes. The study was conducted in accordance with the ethical standards of our institution, while approval of the Institutional Review Board was obtained (Ref. number: 27197, 30/09/2025). Due to the retrospective nature of the study, the requirement for patient consent was waived, while all data were anonymized.

**Figure 1:**
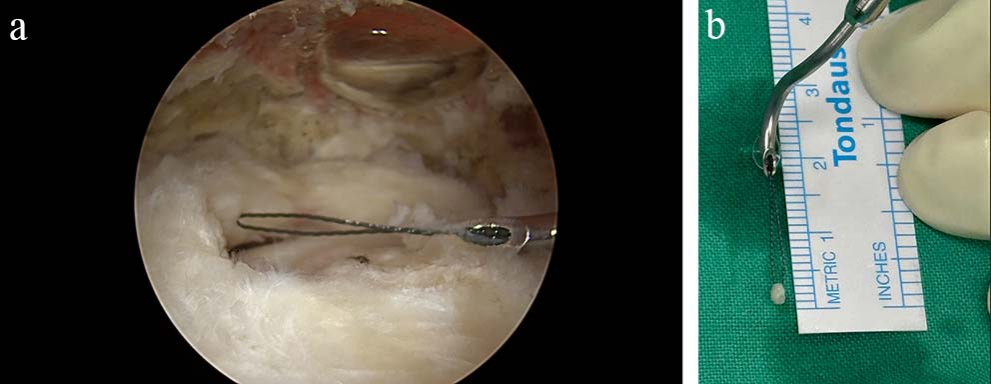
Intraoperative evaluation of the size of the rotator cuff tear (a), by measuring the distance between the most medial edge of the torn tendon and its footprint on the greater tuberosity (b).

Patients were divided into two groups based on intraoperative management of LHBT. In the preservation group, the LHBT remained intact, while in the release group, patients underwent tenotomy of the LHBT despite the absence of pathological lesions. The decision regarding release or retention of LHBT was based on the surgeon’s clinical practice. All procedures were performed by two fellowship-trained shoulder surgeons. One surgeon performed LHBT tenotomy, while the other retained the LHBT. The reconstructive technique for the rotator cuff was the same for both surgeons. During arthroscopy, the intra-articular compartment was initially assessed. The subscapularis tendon and its footprint on the lesser tuberosity were inspected for any lesions, while the whole course of the LHBT was evaluated from its attachment to the glenoid to its insertion into the bicipital groove. The LHBT was evaluated for any lesions, such as tears, fraying of its fibers, or indicative signs of inflammation, such as hypervascularity. In case of LHBT release, the tendon was cut with the use of a radiofrequency ablation close to its attachment onto the glenoid, and the proximal stump was smoothed with an arthroscopic 4.5 mm shaver. Following evaluation of the intraarticular compartment, the subacromial space was accessed. Initially, routine subacromial decompression and acromioplasty were performed using an arthroscopic 4.5 mm shaver and a 5.0 mm burr. Subsequently, the RCT was evaluated, and the size of the tear was measured using a nitinol suture that was inserted through a lasso device. The size of the tear was assessed by measuring the distance between the most medial edge of the torn tendon and its footprint on the greater tuberosity (Figure 1). Afterward, rotator cuff repair was performed using a double-row transosseous equivalent repair technique. Depending on the anteroposterior size of the tear, one or two medial row anchors were used, along with one or two lateral row knotless anchors.

Demographic and clinical data such as age, gender, body mass index, tear size (small, medium, large, massive based on the Codman classification), dominant side, and comorbidities (e.g., diabetes mellitus, smoking) were collected to test the comparability of the groups. Preoperative and postoperative clinical evaluation included assessment of range of motion (ROM) and pain severity by the VAS, while functional results were assessed through the Constant-Murley score and the American Shoulder and Elbow Surgeons (ASES) score. Preoperative evaluation was performed 1 week before surgery, while postoperative evaluation was at 12 and 24 months.

### Statistical analysis

The analysis includes descriptive statistics for the key features of the cohort, while comparisons between the two groups were also performed using the Wilcoxon-rank sum test for continuous variables and the chi-square test for categorical variables. Specifically, the preoperative demographics, tear characteristics, and functional outcomes were compared between the preservation group and the release group. Moreover, to evaluate the independent association between LHBT management and postoperative functional outcomes, and to assess for confounding factors, a multivariable linear regression analysis was performed. In this regression model, the dependent variable was the functional outcome with the independent variables including age, size of RCT, number of anchors, and hand dominance. All analyses were performed using the Stata 19.5 (StataCorp, College Station, TX, USA) software. The statistical significance level was set at *p* < 0.05 for all tests.

## Results

Initially, 257 patients who underwent arthroscopic rotator cuff repair with intraoperative confirmation of normal LHBT were considered eligible for the study. However, 27 patients were excluded due to concomitant pathology, including subscapularis tears (*n* = 11), symptomatic acromioclavicular joint arthritis (*n* = 7), and glenohumeral joint arthritis with chondral lesions of the glenoid and/or humeral head (*n* = 9). Additionally, 7 patients were excluded due to incomplete medical records. Therefore, the final cohort included 223 patients and was finally included in the study. The overall median age of the study population was 62 (interquartile range [IQR]: 56–66) years, while the study population consisted of 118 (52.9%) males and 105 (47.1%) females. Regarding the management of LHBT, the LHBT remained intact in 113 patients (conservation group), while 110 patients underwent release of the LHBT (release group).

The LHBT conservation group and the LHBT release group were comparable in terms of age (medians: 63 vs 60.5, *p* = 0.16), gender (males: 49.5% vs 56.3%, *p* = 0.30), and involvement of dominant arm (92.9% vs 90.0%, *p* = 0.43; Table 1). The two groups were also similar regarding the size of the RCTs (*p* = 0.51). Specifically, small-sized RCTs were evident in 46.0% of patients in conservation group and in 37.3% of patients in the release group, medium -sized RCTs were evident in 36.3% of patients in conservation group and in 40.9% of patients in the release group, while large-sized RCTs were evident in 13.3% of patients in conservation group and in 18.2% of patients in the release group. Repairable massive cuff tears were evident in 5 (4.4%) patients in the conservation group and in 4 (3.6%) patients in the release group. Last, the conservation group and the release group were comparable regarding the number of anchors used for the rotator cuff repair (medians: 2 vs 2, *p* = 0.44; Table 1).

**Table 1:** Demographics and tear size of the study population.

	LHBT conservation group (*n* = 113)	LHBT release group (*n* = 110)	*p*-value
Age (years)	62.3 ± 7.9, 63 (55–57)	60.7 ± 7.1, 60.5 (57–65)	0.16
Gender (males%)	56 (49.5)	62 (56.3)	0.30
Involvement of the dominant arm	105 (92.9)	99 (90.0)	0.43
Tear size			
Small	52 (46.0)	41 (37.3)	0.51
Medium	41 (36.3)	45 (40.9)	
Large	15 (13.3)	20 (18.2)	
Massive	5 (4.4)	4 (3.6)	
Number of anchors for cuff repair	2.1 ± 0.8, 2 (1–3)	2.2 ± 0.9, 2.0 (1–3)	0.44

Abbreviations: LHBT, long head of the biceps tendon.

Data are presented as means ± SD, medians (interquartile ranges), or as absolute frequencies (percentages) when appropriate.

**Figure 2:**
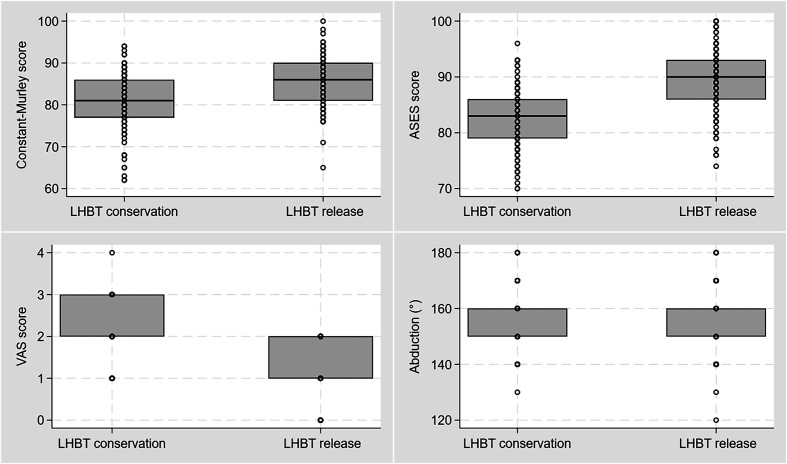
Constant-Murley shoulder score, American Shoulder and Elbow Surgeons (ASES) score, visual analog scale (VAS) score, and range of motion at 24 months following arthroscopic rotator cuff repair and conservation vs. release of the long head of the biceps (LHBT).

Regarding the postoperative results, the median VAS score of patients in the LHBT conservation group was 2 (IQR:2–2) at 12 months and 2 (IQR:2–3) at 24 months postoperatively, while the median VAS score of patients in the LHBT release group was 1 (IQR:0–2) at 12 months and 1.0 (IQR: 1–2) at 24 months postoperatively (Table 2). The VAS score differed between the two study groups at 12 months and 24 months (*p* < 0.001) postoperatively, indicating that patients in the LHBT release group had less pain than those in whom the LHBT was retained (Figure 2). However, the ROM was similar between the two study groups postoperatively. Specifically, shoulder abduction was similar between patients in the LHBT conservation group and those in the LHBT release group at 12 months (medians: 150° vs 150°, *p* = 0.94) and 24 months (medians: 160° vs 160°, *p* = 0.96) postoperatively, while also forward flexion was similar between the two study groups at 12 months (medians: 160° vs 160°, *p* = 0.47) and 24 months (medians: 170° vs 170°, *p* = 0.34) postoperatively (Table 2).

**Table 2:** Pain, range of motion, and functional outcomes for the two study groups.

	LHBT conservation group (*n* = 113)	LHBT release group (Group B, *n* = 110)	*p*-Value
VAS score			
Preoperative	5.8 ± 0.9, 6.0 (5.0–7.0)	6.0 ± 1.0, 6.0 (5.0–7.0)	0.46
12 months	1.9 ± 0.7, 2.0 (2.0–2.0)	1.0 ± 0.7, 1.0 (0.0–2.0)	<0.001
24 months	2.0 ± 0.7, 2.0 (2.0–3.0)	1.0 ± 0.7, 1.0 (1.0–2.0)	<0.001
Abduction (°)			
12 months	153.8 ± 9.5, 150.0 (150–160)	154.0 ± 11.2, 150 (150–160)	0.94
24 months	156.9 ± 10.5, 160 (150–160)	156.9 ± 11.6, 160 (150–160)	0.96
Forward flexion (°)			
12 months	165.9 ± 9.2, 160 (160–170)	164.9 ± 8.8, 160 (160–170)	0.47
24 months	166.5 ± 10.0, 170 (160–170)	167.6 ± 9.1, 170 (160–170)	0.34
Constant-Murley score			
Preoperative	50.0 ± 7.9, 50.0 (44.0–55.0)	50.3 ± 7.9, 50.0 (45.0–55.0)	0.86
12 months	82.0 ± 5.4, 82.0 (79.0–86.0)	82.2 ± 4.5, 83.0 (80.0–85.0)	0.51
24 months	80.8 ± 6.9, 81.0 (77.0–86.0)	85.4 ± 6.0, 86.0 (81.0–90.0)	<0.001
ASES score			
Preoperative	55.2 ± 7.3, 56.0 (51.0–60.0)	54.0 ± 7.9, 54.0 (48.0–60.0)	0.20
12 months	85.6 ± 4.7, 86.0 (82.0–88.0)	86.4 ± 5.0, 87.0 (83.0–90.0)	0.11
24 months	82.6 ± 5.8, 83.0 (79.0–86.0)	89.2 ± 5.7, 90.0 (86.0–93.0)	<0.001

Abbreviations: LHBT, long head of the biceps tendon; VAS, visual analog scale; ASES, American Shoulder and Elbow Surgeons.

Data are presented as means ± SD, medians (interquartile ranges) or as absolute frequencies (percentages) when appropriate.

The preoperative Constant-Murley score was similar between patients in whom the LHBT was retained and those in whom the LHBT was released (medians: 50 vs 50, *p* = 0.86), as well as the ASES score (medians: 56 vs 48, *p* = 0.20). At the 24-month follow-up, the median Constant-Murley score was 81 (IQR: 77–86) for patients in the LHBT conservation group, and 86 (IQR: 81–90) for patients in the LHBT release group (Figure 2). The postoperative Constant-Murley score was higher for those in whom the LHBT was released (*p* < 0.001). In addition, the percentage of patients who experienced a postoperative improvement in the Constant-Murley score above the minimal clinically important difference (MCID) was higher in the release group compared to the conservation group (98.1% vs 92.0%, *p* = 0.034). Regarding the ASES score, the median ASES score at the 24-month follow-up was 83 (IQR: 79–86) for patients in the LHBT conservation group and 90 (IQR: 86–93) for those in the LHBT release group. Similar to the Constant-Murley score, the ASES score was also higher in patients in the LHBT release group (*p* < 0.001), indicating that release of the LHBT resulted in improved functional outcomes compared to LHBT retention in patients who undergo arthroscopic repair of RCTs (Table 2). This finding was also supported by the results of the multiple linear regression analysis, since release of the LHBT (as opposed to LHBT retention) was found to be associated with improved Constant-Murley score (coefficient = 4.85, 95% confidence interval [CI]: 3.13–6.56, *p* < 0.001) and ASES score (coefficient = 6.66, 95% CI: 5.10–8.22, *p* < 0.001) at 24 months postoperatively (Table 3). RCT size was also associated with the functional outcomes, since larger tears were associated with worse Constant-Murley score (coefficient = −1.89, 95% CI: −3.56 to −0.23, *p* = 0.026).

**Table 3:** Results of multivariable linear regression analysis for postoperative outcome as reflected by the Constant-Murley and ASES score as the dependent variable, with age, gender, involvement of the dominant arm, release of the long head of the biceps, rotator cuff tear size, and number of anchors used for the rotator cuff repair as the independent variables.

Variables	Constant-Murley score			ASES score		
	Coefficient	95% CI	*p*-value	Coefficient	95% CI	*p*-value
Age	−0.15	−0.12–0.09	0.77	−0.20	−0.12–0.80	0.69
Gender	−1.14	−2.85–0.57	0.19	−0.44	−2.00–1.10	0.57
Dominant arm	1.41	−1.67–4.49	0.36	1.36	−1.43–4.15	0.33
LHBT release	4.85	3.13–6.56	<0.001	6.66	5.10–8.22	<0.001
Rotator cuff tear size	−1.89	−3.56–−0.23	0.026	−0.52	−2.03–0.98	0.49
Number of anchors used	0.74	−0.80–2.29	0.34	0.59	−0.80–2.00	0.40

Abbreviations: LHBT, long head of the biceps tendon; ASES, American Shoulder and Elbow Surgeons; 95% CI, 95% confidence interval.

## Discussion

The management of LHBT during rotator cuff surgery remains a subject of debate among orthopedic surgeons. While tendon release or tenodesis is widely accepted as a treatment solution when there is obvious pathology, such as degeneration, partial rupture, or instability, the optimal strategy when the tendon appears normal intraoperatively is less clear. Some surgeons prefer release to reduce the likelihood of future pain associated with LHBT, while others advocate retention to avoid a possible decrease in muscle strength and the occurrence of complications. The findings of this study indicate that routine LHBT release during rotator cuff repair is associated with less postoperative pain, which may imply a progressive degeneration of the LHBT, although the tendon may appear macroscopically intact during arthroscopy. Most importantly, the observed difference in Constant-Murley score between the two groups was not only statistically, but also clinically important, since there was a higher percentage of patients who reported an improvement higher than the MCID in the release group compared to the retention group. However, long-term available data on the functional consequences of releasing a normal LHBT are limited, and further study is needed.

The superior functional and pain outcomes observed after routine LHBT tenotomy may be attributed to the removal of an occult pain generator, even when the tendon appears macroscopically intact. Degenerative and inflammatory alterations in the LHBT have been reported histologically in up to 50% of apparently normal tendons, suggesting that visual inspection by arthroscopy alone may underestimate tendon pathology [10]. Histologic alterations include mucoid degeneration, destruction, and disorientation of collagen fibers, as well as necrosis of the tendon tissue [10]. These microscopic alterations can progress to macroscopic lesions and cause chronic pain and discomfort. Routine tenotomy could therefore prevent this progression, which may explain the lower VAS scores in the LHBT release group compared to the LHBT retention group. Biomechanically, the LHBT has a more prominent role as an anterior shoulder stabilizer and humeral head suppressor in pathologic conditions such as RCTs than it does in healthy shoulders [2, 11]. Rotator cuff deficiency may be evident postoperatively despite rotator cuff repair; therefore, the LHBT may be hyperactivated to compensate for instability, leading to progressive tendinopathy from friction [2, 5, 11]. This postoperative tendinopathy may be even more prominent in cases of rotator cuff re-tears, leading to shoulder pain. The varying degree of postoperative LHBT tendinopathy may explain why rotator cuff re-tears are not always associated with shoulder symptoms. Routine tenotomy prevents this progression without a significant ROM deficit. Furthermore, retaining a pathological tendon may be disadvantageous since repair of the subscapularis or supraspinatus during RCR negatively influences LHBT movement in the bicipital groove. Biceps tenotomy/tenodesis could be a solution to this problem [5]. According to Veen et al., an isolated LHBT tenotomy improves pain and shoulder functionality in degenerative RCT patients 4 years after surgery [12]. This could be a viable option for patients with normal acromiohumeral distance and failed conservative treatment [12].

However, many surgeons argue against a routine LHBT release during RCR. Clinically, tenotomy has been associated with higher rates of aesthetic deformity (i.e., Popeye sign), anterior shoulder pain, subjective weakness, and cramping compared to tenodesis [13]. Their frequency is estimated at 14.1%, 7.8%, 10.4% and 10.4% respectively [14]. Since these complications are associated with younger, male patients and with workers’ compensation injuries, LHBT retention could be reasonable in those cases [14]. In our study, the superior functional outcomes of the tenotomy group indicate that complications such as anterior shoulder pain and cramping were insignificant in our cohort. However, aesthetic outcomes were not assessed in our study, while we only included older patients with degenerative RCTs (i.e., >50 years old).

Despite the clinical importance of the topic, the available literature is limited. Most original studies and meta-analyses focus on the postoperative outcomes of adjunctive LHBT management (tenotomy or tenodesis) in patients with RCTs and concomitant LHBT lesions [7, 15, 16]. A recent retrospective study by Malavolta et al. showed no significant difference in postoperative ASES and University of California at Los Angeles (UCLA) scores between patients undergoing isolated RCR and those undergoing RCR with LHBT surgery [17]. However, in this study, LHBT surgery was indicated by tendon subluxation, dislocation, >25% thickness tear, and type 2 or 3 SLAP lesions. Therefore, tenotomy or tenodesis was not routinely performed [17]. Godeneche et al. also compared RCR with and without LHBT lesions 10 years after surgery [7]. Patients with LHBT lesions were subjected to adjunctive biceps tenotomy or tenodesis, whereas patients with normal tendons were subjected to isolated RCR. In their retrospective study, the authors found no significant differences between the two cohorts in postoperative constant and SST scores, as well as rates of supraspinatus retears [7]. Therefore, the authors oppose the use of routine LHBT surgery [7]. Although the findings of these studies may contradict our results, their inclusion criteria differ fundamentally from our study since patients were subjected to tenotomy or tenodesis in cases of LHBT pathology instead of routinely.

Similarly, Kawashima et al. also advocate the benefits of LHBT retention in younger patients (i.e., <55 years old) with medium or small-sized tears [18]. Their conclusion draws largely on ultrasonographic assessment of LHBT hypertrophy and vascularity in the affected shoulder, compared to the contralateral healthy shoulder. According to their study, no significant LHBT hypertrophy was evident in the affected shoulder, compared to the contralateral healthy ones [18]. Moreover, while there was no difference between pre- and postoperative vascularity of the bicipital groove, patients with increased vascularity reported significantly lower pain scores, compared to patients with negative vascularity [18]. Therefore, ultrasonography could contribute to decision-making for younger patients with small to medium RCTs. In our study, only patients aged 50 years or older were considered eligible for inclusion. Therefore, increased vascularity may be unlikely in older patients, which may explain why the LHBT release group reported superior functional scores compared with the tendon retention group. Admittedly, the differences in study population, inclusion criteria, and postoperative patient assessment contribute to the heterogeneity of the literature.

This study is not without limitations. First, the retrospective nature of the study may limit the generalizability of the results. Prospective randomized controlled trials are required to validate our findings and define a more robust clinical importance. Although there is potentially selection bias due to the retrospective nature of the study, the baseline characteristics of the two groups were comparable without statistically significant differences. Moreover, although our study provides solid mid-term outcomes of routine LHBT release, a longer follow-up period would strengthen our results and enable assessment of functional improvement or decline over time. Furthermore, muscle strength was only assessed in the context of the Constant-Murley score, and no separate analysis was performed. It is therefore unclear whether routine LHBT leads to significant muscle strength deficit compared to isolated RCR. Aesthetic complications, i.e., Popeye deformity, were also not assessed. However, literature reports no significant muscle weakness or aesthetic complications in elderly patients with LHBT tenotomy or proximal tendon rupture [19, 20]. Lastly, no histological examination of the excised tendon was performed, and the tendon’s microscopic status remains unclear. Despite these limitations, our study provides useful insight into a polarizing clinical topic that is poorly explored in the literature.

## Conclusions

In conclusion, our study supports the notion that routine tenotomy of the LHBT provides functional benefits for degenerative RCT patients, without significant limitations in active ROM. Further research should include prospective randomized studies, focusing on routine and selective tenotomy compared to isolated RCR. Imaging studies may also provide useful insight into disease progression and tendon integrity.

## Data Availability

Data associated with this article cannot be disclosed due to ethical reasons.
